# Differences in fear response strategy and stress susceptibility amongst four different commercial layer strains reared cage free

**DOI:** 10.3389/fphys.2022.943471

**Published:** 2022-08-16

**Authors:** Austin A. Brown, Eric B. Sobotik, Gabrielle M. House, Jill R. Nelson, Gregory S. Archer

**Affiliations:** Department of Poultry Science, Texas A&M University, College Station, TX, United States

**Keywords:** fear, stress, layer, cage free, variation

## Abstract

Different commercial lines of laying hens may show varying levels of fearfulness in response to stressful events or situations. It is important to understand the differences in fear response and stress susceptibility. In this study, four commercial laying hen lines reared from hatch to 32 weeks of age in a cage free system Strains consisted of a brown egg laying line (Hyline Brown; HB) and three white egg laying lines (W36, W80, and LSL). Sixty hens from each strain were used. Each hen was assessed for fearfulness using the following tests: isolation (ISO), emergence (EMG), inversion (INV), and tonic immobility (TI). Stress was assessed based on physical asymmetry (ASYM), corticosterone (CORT) concentrations, and heterophil:lymphocyte ratio (HL). At 3 weeks of age, the W80 birds exhibited more vocalizations during ISO and a shorter duration to emerge than other lines except the HB birds during EMG. Conversely the W36 birds had fewer vocalizations during ISO and emerged quicker than other birds except the LSL during EMG. At 16 weeks of age, the LSL and the W36 bird demonstrated greater fear in TI than the HB. At 30 weeks of age, the observed fear response strategies of each strain changed from previous age and differences were observed between lines (*p* < 0.05). At both 16 and 30 weeks of age the HB birds had the highest (*p* < 0.05) stress indicators (CORT, HL, and ASYM). Furthermore, they had a higher CORT after acute stressor (*p* < 0.05). Commercial lines of laying hens show clear variation in their stress response strategy and stress susceptibility. Brown egg laying hens tend to actively avoid perceived threats whereas white egg laying hens use passive avoidance. Brown egg laying hens also have higher levels in the measures of stress susceptibility than white egg laying hens. Understanding of individual strain response to fearful stimuli and other stressors is important knowledge to appropriately determine welfare differences between strains of layers as the baseline measures are often different.

## 1 Introduction

One of the primary principles of optimum animal welfare is minimal fear and stress. One way to insure these are minimized is to select the appropriate animals for the housing system that they will be reared in. With the current transition from traditional cage housing towards alternative cage free housing worldwide in the laying hen industry this is becoming a huge concern. Many commercial strains were selected to cope with living in cages and simply switching them to a cage free system may not be optimal for their welfare as doing so could result in excessive fear and stress. As excessive or prolonged fear in animals can result in wasted energy, injuries, behavioral inhibition, reduced ability to adapt to change, delayed maturation, decreased growth and reproduction, and death (Jones, 1996) it should be avoided. Furthermore, fearfulness in laying hens can even result in severe feather pecking behavior (Agnvall et al., 2012; de Hass et al., 2013) and broken keel bones (Harlander-Matauschek et al., 2015) further decreasing their welfare.

Fear responses can be classified as either passive avoidance (freezing, tonic immobility), or active avoidance (withdrawal, fighting, and vigorous escape) (Jones, 1987). The freezing and tonic immobility associated with passive avoidance behavior and the fighting and fleeing associated with active avoidance behavior have been classified as the main four types of anti-predator fear related responses (Ratner, 1967). These anti-predator behaviors have been demonstrated to be the most reliable when evaluating fear (Miller et al., 2006). Previous research has demonstrated fear responses in laying hens have heritability ranging from very low at 0.07 to moderate at 0.49 (Uitdehaag et al., 2011; de Haas et al., 2013; Grams et al., 2015), consequently making fear-related behavioral responses a selectable trait in poultry.

Stress susceptibility is closely related to fear responsiveness, meaning that lower physiological indicators of stress susceptibility are also reliable indicators of overall animal welfare. When a bird becomes stressed, the hypothalamic-pituitary-adrenal axis is activated, ultimately resulting in the secretion of corticosterone (CORT) into the bloodstream (Mormede et al., 2007; Virden and Kidd, 2009). As the primary stress hormone in birds, excessive CORT threatens bird health by suppressing immune responses (Beard and Mitchell, 1987), altering metabolism to increase readily available energy (Mormede et al., 2007), slowing growth rate (McFarlane et al., 1989), and disrupting cecal microflora (Burkholder et al., 2008). Therefore, the most common way to measuring stress is by measures corticosterone levels in the blood (Mormede et al., 2007). Excessive corticosterone concentrations lead to hypotrophy of the lymphoid organs which results in a higher heterophil/lymphocyte ratio, which can be used as an indicator of long-term stress (Gross and Siegel, 1983). Physical asymmetry, which is a simple comparison of the growth of bilateral structures on a bird (Campo et al., 2008), has been strongly correlated to stress susceptibility (Graham et al., 1993; Knierim et al., 2007; Archer and Mench, 2013) and can be used in determining a bird’s ability to cope with stressors to that point of life (Kellner and Alford, 2003).

Breed differences in fearfulness and stress susceptibility have been reported in previous research (Albentosa et al., 2003; Welfare Quality, 2009; Abe et al., 2013; Ferrante et al., 2016; Giersberg et al., 2020; Peixoto et al., 2020; Wei et al., 2022). However, Anderson and Jones (2012) observed no differences in tonic immobility among four genetic lines of White Leghorn hens although basal plasma corticosterone concentration differed between lines. Complicating the matter even more, it has been demonstrated that differences may be seen in one fear test but not in others (Albentosa et al., 2003). While Archer (2018) observed differences between different strains of fowl it was not always consistent across type of fear test. Archer (2018) did, however, observe that even within White Leghorns fear responses differed between different genetic lines selected for characteristics not related to fear and stress. Nelson et al. (2020) observed that brown egg layers and white egg layers differed in fear and stress responses over a large variety of measures. They concluded that brown egg laying hens tended to actively and passively avoid predators or threats. It should be noted that many of these previous research projects did not document or did not rear or test the strains in the same environment. Furthermore, many studies tested the birds either when they were housed in cages during production or in mismatch production systems.

The objective of this study was to determine how different commercial layers responded during fear tests and stress measures at different ages while being reared in a cage free system. Based on previous literature it was hypothesized that there would be differences in fear and stress measures between white and brown egg layers and possibly between white leghorn varieties. Evaluation and understanding of these differences will allow for the selection of the optimal commercial birds for different housing systems or at least for producers to be aware of the challenges some strains may have compared to others.

## 2 Materials and methods

### 2.1 Animals and husbandry

Sixty birds from each of four different genetic strains—three White Leghorn strains (Hyline W36, Hyline W80, and Lohmann LSL) and one brown layer strain (Hyline Brown)—were used in this study. All birds were grown from day of hatch until 32 weeks of age. All birds were obtained from the same commercial hatchery and were not beak treated. Each strain was reared in one of four floor pens (3.05 × 4.57 m) on wood shaving. All pens were adjacent and all environmental conditions were managed equally according to the guidelines set forth in the Guide for the Care and Use of Agricultural Animals in Research and Teaching (FASS, 2010) and all procedures were approved by the Texas A&M university animal care and use committee (IACUC 2017-0259). Nest boxes and perches were provided to all pens. Fear tests and physiological stress was measured at several time points during the 32-week experiment as described below. All birds were tested in all measures collected. Testing occurred in the space between animal home pens.

### 2.2 Fear measurements

#### 2.2.1 Isolation (ISO)

This test was modified from methods outlined in (Archer and Mench, 2014). The isolation tests were performed at 3 weeks of age. Prior to testing 15 chicks were caught from a strain, placed in a holding area and then tested. This was repeated for each strain and the process was repeated until all birds had been tested. Order of strain was randomized for each testing block. The birds were individually placed in an unlidded 19 L bucket. A timer was set for 3 min, and the number of vocalizations produced by the bird during this time was counted. More vocalizations were considered to indicate more fearfulness (Forkman et al., 2007).

#### 2.2.2 Emergence (EMG)

The emergence test was conducted at 3 weeks of age, modified from methods found in Archer and Mench (2014). A lidded 19 L bucket was modified to have a sliding door in the side, and the person performing the test was seated behind the door and not visible to the bird. A video camera and monitor were used to see when the bird emerged from the container. Prior to testing 15 chicks were caught from a strain, placed in a holding area and then tested. This was repeated for each strain and the process was repeated until all birds had been tested. Order of strain was randomized for each testing block. The birds were individually placed in the bucket with the door and lid closed. After 20 s, the door was slid open and a timer was started. The timer was stopped when the bird first stepped out of the container into the shaving lined testing pen, or at a maximum of 3 min. Longer latency to emerge was considered to indicate more fearfulness (Archer and Mench, 2014).

#### 2.2.3 Tonic immobility (TI)

The test was carried out on all birds as described by Archer and Mench (2014) at 16 and 30 weeks of age. Prior to testing 15 chicks were caught from a strain, placed in a holding area and then tested. This was repeated for each strain and the process was repeated until all birds had been tested. Order of strain was randomized for each testing block. Each block was tested on 1 day at the same time of day for each testing period. In brief, each bird was placed in a u-shaped cradle on its back and head covered for 10 s then released and time to first head movement were recorded by an observer that sat 1 m away. If the bird could not be induced in three attempts it was scored as zero. Latency to first head movement, latency to right and number of induction attempts was recorded. The test was terminated in 600 s if a bird failed to right, and that bird was scored as 600. Longer latency to right indicated greater level of fear.

#### 2.2.4 Inversion (INV)

At 16 and 30 weeks of age all birds were also subjected to (INV), as described by Newberry and Blair (1993) and Archer and Mench (2014). Prior to testing 15 chicks were caught from a strain, placed in a holding area and then tested. This was repeated for each strain and the process was repeated until all birds had been tested. Order of strain was randomized for each testing block. Each block was tested on 1 day at the same time of day for each testing period. Each bird was removed from its crate and then inverted by its legs using one hand until the bird ceased to flap its wings, or for a maximum of 30 s. Flapping intensity was determined using the number and duration of wing flaps, and a higher intensity indicated greater level of fear.

### 2.3 Stress measures

#### 2.3.1 Plasma corticosterone (CORT)

Basal corticosterone was determined at both 16 and 31 weeks of age. Acute stress response was determined at 30 weeks of age. All blood samples were taken between 0800-1,000. To do this all birds were placed in transport crates in groups of 20 for 1 h then blood was collected procedures described by Archer and Mench (2013). At each time point approximately 1–2 ml of blood was collected from each bird *via* the wing vein within 1 min of being caught from home pen. Plasma was then stored and analyzed as described in Nelson et al. (2018). Total plasma corticosterone concentrations were determined using a 96-well commercial ELISA kit (ADI-901-097, Enzo Life Sciences, Inc., Farmingdale, NY). Absorbance was read at 450 nm using a microplate absorbance reader (Tecan Sunrise, Tecan Trading AG, Switzerland). Intra and inter plate %CV was less that 5%.

#### 2.3.2 Heterophil to lymphocyte ratio (HL)

The heterophil to lymphocyte ratio was conducted using the method described in Nelson et al. (2018) at 16 and 30 weeks of age. Dry blood smear slides were stained with a neat stain hematology stain kit (Cat. #25034, Poly Sciences, Inc., Warrington, PA), and used to determine H/L ratio at 40x magnification using an oil immersion lens under microscopy (89404-886, VWR International, Radnor, PA).

#### 2.3.3 Physical asymmetry (ASYM)

Physical asymmetry score for all birds was assessed following the protocol outlined in Archer and Mench (2013) at 16 and 30 weeks of age. Using a calibrated Craftsman IP54 Digital Caliper (Sears Holdings, Hoffman Estates, IL), the middle toe length, metatarsal length, and metatarsal width were measured for both the right and left legs. Composite asymmetry score was calculated by taking the sum of the absolute value of left minus right of each trait, then dividing by the total number of traits. Thus, the formula for this trial would be: Composite Asymmetry Score=(|L−R||MTL+|L−R|ML+|L−R|MW)÷3

### 2.4 Statistical analysis

All data was analyzed using the GLM procedure as used with strain, age, and strain × age interaction as the model apart from ISO, EMRG, and acute stress CORT data which were analyzed using ANOVA for strain effects only. The assumptions for ANOVA were tested using Shapiro-Wilk test for normality and Levene’s test for homogeneity of variance. All analyses were performed using SAS 9.3 for Windows (SAS Institute Inc., Cary, NC). Data that did not meet the assumptions for ANOVA was analyzed using the Kruskal–Wallis test on the equality of the medians, adjusted for ties. When significant differences were found, the Dwass-Steel-Critchlow-Fligner method (Hollander and East, 1999) was used to test for all possible comparisons. Significant differences were defined as *p* < 0.05.

## 3 Results

### 3.1 Fear measures

As shown in Table 1, differences among strains were observed for all measures of fear. At 3 weeks of age, the W80 birds vocalized more (59.00 ± 7.94) than all other lines of layers (*p* < 0.05) and the W36 birds vocalized the least (12.25 ± 3.39) compared to all other lines (*p* < 0.05) during ISO. The LSL (37.28 ± 6.58) and HB (31.27 ± 4.40) birds were intermediate. Both the W80 (15.37 ± 3.98 s) and the HB (13.65 ± 2.55 s) birds emerged faster (*p* < 0.05) during the EMG than the W36 (40.67 ± 6.83 s) and LSL (39.12 ± 7.29 s) birds.

**TABLE 1:** Fear response of four strains of commercial layers (W36, W80, LSL, and Hy-Line Brown (HB)) at 3, 16, and 30 weeks of age.

Test	Time point	Measurement	W36	W80	LSL	HB	SEM
Isolation	3 weeks of age	Vocalizations	12.25^c^	59.00^a^	37.28^b^	31.27^b^	3.10
Emergence	3 weeks of age	Time (sec)	40.67^a^	15.37^b^	39.12^a^	13.65^b^	2.87
Tonic Immobility	16 weeks of age	Head (sec)	37.10^b^	37.00^b^	108.60^a^	5.80^c^	6.12
		Right (sec)	333.3^a^	240.8^b^	318.6^a^	176.0^b^	13.0
		Difference (sec)	296.2^a^	203.8^b^	210.0^b^	170.2^b^	12.2
		Attempts	1.15	1.17	1.12	1.33	0.03
	30 weeks of age	Head (sec)	14.23^a^	6.97^ab^	8.32^ab^	4.22^b^	1.50
		Right (sec)	262.6	270.9	264.0	336.5	13.9
		Difference (sec)	248.4^b^	263.9^ab^	255.7^ab^	332.3^a^	13.9
		Attempts	1.15	1.30	1.13	1.28	0.03
	Inversion	16 weeks of age	Flaps	69.53^a^	55.80^b^	56.18^b^	61.93^ab^	1.67
Time (sec)			11.08	9.98	10.57	10.15	0.26
Intensity (flaps/sec)			6.14^a^	5.56^b^	5.60^b^	6.15^a^	0.09
30 weeks of age		Flaps	41.50^b^	48.15^ab^	55.30^a^	56.97^a^	1.79
		Time (sec)	8.53	9.57	10.17	9.12	0.33
		Intensity (flaps/sec)	4.45^days^	5.10^c^	5.67^b^	6.28^a^	0.10

Different superscripts within row indicate differences between layer lines (*p* < 0.05).

There was a strain effect observed in latency to first head movement during TI (*p* = 0.01). The HB strain had the shortest time to first head movement with the LSL having the longest latency and the other two strains being intermediate. No effect of age nor an interaction was observed in latency to first head movement during TI (*p* > 0.05). No overall difference was observed between strains (*p* = 0.20) or age (*p* = 0.35) in latency to right during TI. However, there was an interaction observed in latency to right (*p* < 0.001). No overall strain effect was observed in the difference in time from first head movement to righting (*p* = 0.33). An age (*p* = 0.002) and interaction (*p* = 0.001) effect was observed in the difference in time from first head movement to righting. No differences were observed between strains, age nor interaction of the two in number of attempts to induce TI (*p* > 0.05).

There was no strain effect observed in number of flaps during INV (*p* = 0.19). There was an age effect (*p* < 0.001) and interaction (*p* < 0.001) in number of flaps during INV. More flaps during INV were observed at 16 weeks than at 30 weeks. There was no strain (*p* = 0.63) or interaction (*p* = 0.24) effect observed in duration of flapping during INV. There was an effect of age on duration of flapping during INV (*p* = 0.01) with durations being longer at 16 weeks of age compared to 30 weeks. There was an effect of strain (*p* < 0.001), age (*p* < 0.001) and an interaction (*p* < 0.001) on intensity of flapping during INV. The HB strain had greater flapping intensity during INV than all other treatments. Intensity was observed to greater at 16 weeks compared to 30 weeks.

At 16 weeks of age, the HB had the shortest latency to first head movement during TI (5.80 ± 2.80 s, *p* < 0.05) compared to all other lines. The LSL had the longest latency to first head movement during TI (108.60 ± 16.10 s, *p* < 0.05) compared to all other lines (*p* < 0.05). W36 and W80 were intermediate in latency to first head movement during TI. The W80 (240.8 ± 24.4 s) and HB (176.0 ± 22.3 s) birds had shorter latency to right during TI compared to both the W36 and LSL birds (333.3 ± 26.4 s and 318.5 ± 26.2 s, respectively, *p* < 0.05). The W36 birds had a greater difference from first head movement to righting than all other lines (296.2 ± 27.8 s, *p* < 0.05). No differences were observed in number of attempts to induce TI (*p* > 0.05). The W36 birds flapped more (69.53 ± 4.66, *p* < 0.05) than the W80 and LSL birds during INV (55.80 ± 2.74and 56.18 ± 3.17, respectively) with the HB birds (61.93 ± 2.18) being intermediate. No differences were observed in duration of flapping during INV (*p* > 0.05). The W36 and HB birds flapped more intensely (6.14 ± 0.12 flaps/sec and 6.15 ± 0.09 flaps/sec, respectively, *p* < 0.05) than the W80 and LSL birds (5.56 ± 0.13 flaps/sec and 5.60 ± 0.31 flaps/sec, respectively) during INV.

At 30 weeks of age, the W36 had a longer latency to first head movement during TI (14.23 ± 4.12 s, *p* < 0.05) than the HB birds (4.22 ± 1.82 s) with the W80 and LSL being intermediate. No differences in time to right were observed at this time point (*p* > 0.05). The HB birds had a greater difference from first head movement to righting (332.3 ± 29.9 s, *p* < 0.05) than W36 birds (248.4 ± 26.1 s) with the W80 and LSL birds being intermediate. No differences in attempts to induce TI were observed (*p* > 0.05). The LSL and HB birds flapped more (55.30 ± 3.47 and 56.97 ± 2.60, respectively, *p* < 0.05) than the W36 birds (41.50 ± 4.12) during INV with the W80 being intermediate. No differences were observed in duration of flapping during INV (*p* > 0.05). All lines differed from each other in intensity of flapping during INV (*p* < 0.05) with the order going from least to most intense as follows: W36, W80, LSL, HB.

Additionally, to the treatment differences observed within the age timepoints there were some differences within strains at the two ages. The HB strain increased in righting time during TI from 16 to 30 weeks of age, while the other strains showed no differences between ages. The difference between first head movement and righting time was greater in HB birds at 30 weeks compared to 16 weeks as well while the W36 strain had the opposite trend, and the other two strains did not differ between time points. Only the W36 strain demonstrated an increase in flapping number from 16 to 30 weeks of age, all others were equal. Both the W36 and W80 strains demonstrated an increase in flapping intensity from 16 to 30 weeks of age, the other two strains were equal.

### 3.2 Stress measures

Data for plasma CORT, HL and ASYM, is shown in Table 2. There was no age effect on corticosterone concentrations over all (*p* = 0.337). An effect of strain was observed with HB having higher overall CORT concentrations than all other strains (*p* < 0.001). There was also an interaction effect observed between strain and age (*p* = 0.008). There was an effect of strain (*p* < 0.001) on HL with the HB strain having the highest HL and the having the W80 strain having the lowest HL and the other two strains being intermediate. There was also an effect of age on HL with the 30 week sampling having higher (*p* < 0.001) HL than the 16 week sampling. An interaction of strain and age was also observed in HL (*p* < 0.001). There was an effect of strain on ASYM (*p* = 0.048) with the HB strain having higher scores than the W80 and the LSL strains and the W36s being intermediate. There was no effect or age nor an interaction effect on ASYM (*p* > 0.05).

**TABLE 2:** Stress susceptibility measures of four commercial strains of layer chickens (W36, W80, LSL, and Hy-Line Brown (HB)) at 16 and 30 weeks of age and after an acute stress test.

Measure	Time point	W36	W80	LSL	HB	SEM
Plasma Corticosterone (ng/dl)	16 weeks of age	11.65^b^	11.72^b^	14.71^b^	23.34^a^	1.17
	30 weeks of age	16.90^ab^	14.09^b^	17.51^a^	17.51^a^	0.55
	31 weeks of age acute stressor	16.67^b^	16.09^b^	21.36^a^	19.06^a^	0.77
Heterophil: Lymphocyte Ratio	16 weeks of age	0.121^b^	0.062^c^	0.108^bc^	0.178^a^	0.001
	30 weeks of age	0.318^b^	0.194^b^	0.272^b^	0.588^a^	0.028
Composite Asymmetry Score	16 weeks of age	1.533^ab^	1.353^b^	1.196^b^	2.071^a^	0.126
	30 weeks of age	1.468	1.474	1.394	1.636	0.079

Different superscripts within row indicate differences between layer lines (*p* < 0.05).

At 16 weeks of age, the HB birds had the highest plasma CORT concentrations (23,340 ± 1924 pg/dl) compared to all other lines (average 12,695 ± 2,177 pg/dl, *p* < 0.05). The W80 had the lowest HL ratio (0.062 ± 0.011) compared to W36 (0.121 ± 0.019, *p* = 0.02) and HB birds (0.178 ± 0.020, *p* = 0.001) with the LSL (0.108 ± 0.020) being intermediate. The HB birds also had the highest composite ASYM (2.071 ± 0.450) compared to both the W80 (1.353 ± 0.144, *p* = 0.04) and LSL (1.196 ± 0.096, *p* = 0.01) with the W36 (1.533 ± 0.142) being intermediate.

At 30 weeks of age, the HB (17,509 ± 1,216 pg/dl, *p* = 0.03) and LSL birds (17,508 ± 980 pg/dl, *p* = 0.03) had higher plasma CORT compared to W80 birds (14,092 ± 1,279 pg/dl) with the W36 birds (16,903 ± 696 pg/dl) being intermediate. The HB birds had the highest HL ratio compared to all other lines (*p* < 0.05). No differences in composite ASYM were observed at this time point between any of the layer lines.

The HB birds CORT concentrations decreased from the 16 week sampling when compared to the 30 week sampling. While the W36 birds CORT concentrations increased from 16 to 30 weeks and the other two strains had no differences between time points. All strains had an increase in HL from 16 to 30 weeks of age. No difference was observed in ASYM from 16 to 30 weeks of age.

Following the acute stressor at 31 weeks of age, the LSL birds had higher plasma CORT concentrations (21,358 ± 1,367 pg/dl) than both the W36 (16,668 ± 1,254 pg/dl, *p* = 0.03) and W80 birds (16,092 ± 1982 pg/dl, *p* = 0.02) with the HB birds (19,059 ± 1,226 pg/dl) being intermediate.

## 4 Discussion

All layer lines in this current study were reared from day of hatch until the end of the study in a cage free system. Not only did different lines show differing levels of fear during the fear tests in the current study, they also exhibited what could be concluded as differing fear response personality strategies. Jones et al. (1987) stated that fear responses could be classified as either passive avoidance (freezing, tonic immobility), or active avoidance (withdrawal, fighting, and vigorous escape). Furthermore, fear has been suggested as a personality trait in a variety of animal species (Gosling, 2001). At 3 weeks of age, W80 birds exhibited more vocalizations during ISO and a shorter duration to emerge than other lines except the HB birds during EMG. Conversely, the W36 birds had fewer vocalizations during ISO and emerged quicker than other birds except LSL birds during EMG. These results indicate that W80 birds at this age were more active avoiders while the W36 were more passive avoiders. The W80 birds by vocalizing and not freezing demonstrated that they were actively avoiding. By not vocalizing or freezing longer, the W36s demonstrated a more passive avoidance fear response. The LSL birds, while not as evident as the W36 birds, also took more of passive response while the HB birds took a more active response at this age. At 16 weeks of age the results of this current study indicated similar trends in fear response strategies for each line of hen. The LSL birds at this age demonstrated a more passive avoidance strategy in both TI and INV than other lines. The W36 birds had a more mixed fear response with more active avoidance during INV but more passive avoidance during TI. The W80 birds were more intermediate, not appearing to be more active or passive than other lines. The HB birds at this age were generally more active avoiders than other strains. At 30 weeks of age, the observed strategies of each strain when compared to the others changed from previous ages for most of lines. The W36 birds remained somewhat mixed in their fear strategy response. At this age, W36 birds did not flap the most as they did at 16 weeks of age, but they still flapped the most intensely. Similarly, W36 birds at 30 weeks of age had the shortest difference from first head movement until righting rather than the shortest difference for this measure. The W80 birds remained intermediate with their fear response strategy, while LSL birds shifted to a more active strategy at 30 weeks as indicated by more flapping and increased flapping intensity compared to other lines in INV.

Similar to this current study Nelson et al. (2020) observed that brown egg laying lines demonstrated more active fear strategies than white egg laying lines. In the current study birds were housed cage free while in Nelson et al. (2020) birds were housed in cages, making the consistent results found in each in fear strategies profound. Generally, the brown egg layers were active fear responders in both studies demonstrating possibly that system does not affect their strategy. While white egg layers may be affected by their rearing environment as the birds in this current study did exhibit some more active strategies, although further investigation is required. In the current study, W36 and HB birds flapped more intensely than W80 and LSL birds at 16 weeks of age; however, at 30 weeks of age, W36 birds flapped the least intensely of all the strains while HB flapped the most intensely of all strains. The results for W36 birds coincide with previous results by Albentosa et al. (2003), which reported a reduction in fearfulness between 4 and 12 weeks of age for various strains of laying hens. Although the fear responses for the HB birds in this current study do not follow the results of Albentosa et al. (2003), it does support the idea that strains differ in their responsiveness. As demonstrated in previous research (Albentosa et al., 2003; Welfare Quality, 2009; Abe et al., 2013; Ferrante et al., 2016; Archer, 2018; Nelson et al., 2020) even layer hen lines within a breed like white leghorns can differ in their fear response.

At both 16 and 30 weeks of age the HB birds had the highest stress indicators (CORT, HL, and ASYM). Furthermore, they had a higher CORT after acute stressor. The white egg laying hens didn’t differ from each other in stress measures generally with the exception of the W80 birds having the lowest HL at 16 weeks of age of all the strains in this current study and the LSL birds having higher CORT at 30 weeks of age and after acute stressor than the other two white egg layer strains. These results are consistent with results observed by Nelson et al. (2020) where brown egg layers had higher stress indicators than white egg layers. These results could mean that brown egg layers either have higher stress susceptibility or that their basal levels of corticosterone are higher than white egg layers. Both conclusions could greatly impact the selection of what line to house in a certain system or even how comparing strains is possible. Determining which is the case requires future research. Though it has previously been demonstrated that different strains chickens have different basal corticosterone concentrations (Decuypere et al., 1989) therefore comparing direct CORT concentrations between strains may not be as useful as the change in response to stressors. Pusch et al. (2018) found some contradictory findings to this current study. However, their study birds were housed individually in cages prior to and during testing which likely greatly affected the birds’ responses. Furthermore, the stress measures seem to be more stable over time than fear responsiveness which may indicate as the birds age and experience more they may change their fear response strategy. It has also been demonstrated that environmental effects like lighting or maternal stress (Peixoto et al., 2020; Peixoto et al., 2021) can cause differential effects in fear and response of offspring of those birds in different layer strains.

Selecting the appropriate animals for specific housing types is paramount to ensuring optimal welfare. Fear and distress are two major factors that can be detrimental to animal welfare. Fearful animals tend to be less productive (Lyons, 1989; Hemsworth et al., 1990; Hemsworth et al., 1994; Voisinet et al., 1997; Minvielle et al., 2002) in addition to having compromised welfare. Both genetics (Craig et al., 1983; Jones et al., 1991; Mills and Faure, 2000) and developmental experiences appear to determine the propensity for fearfulness within individual birds (Boissy, 1995). Fear and stress are not synonymous; however, fear encompasses many biological systems that mediate the physiological stress response in poultry (Jones, 1986). Prolonged, severe stress causes significant biological damage *via* a cascade of behavioral, physiological, and immunological actions that divert energy away from normal biological functions (Cockrem, 2007; Lambert, 2009).

Often poultry and other livestock are selected for production characteristics without any emphasis or consideration of behavioral characteristics. Although modern domestic flocks do not experience predation from the same threats their wild predecessors, the innate emotion of fear still persists in poultry, and are often redirected from classic predators, such as hawks, to human handlers and environmental changes (Boissy, 1995). Selective breeding of less fearful individuals has been suggested as a means for improving both animal welfare and, in turn, productivity (Fordyce et al., 1988; Hemsworth et al., 1990; Mills and Faure, 1990; Manteca and Deag, 1993; Jones and Hocking, 1999). Historically, white egg laying lines have been housed in cages and brown egg layers in cage free housing. However, currently there is pressure to transition to predominately cage free housing, making it important to understand the differences in fear responsiveness of various layer lines in order to select the best lines to rear in each setting and also optimize the management of all layer lines.

## 5 Conclusion

In conclusion, this study confirmed that different lines of laying hens have varied fear responses and stress susceptibly. With the shift of the predominant commercial housing system to cage free it will become more important to understand how existing and future commercial lines of laying hen respond to fearful situations and to stressors. Selecting for these parameters in addition to production parameters will be paramount to ensure optimal bird welfare. Furthermore, the current study demonstrated that while stress and fear measures are useful tools for welfare assessment, a comparison of birds across genetic strains may be confounded by differences in CORT basal concentrations or innate fear response strategy.

## Data Availability

The original contributions presented in the study are included in the article/Supplementary Materials, further inquiries can be directed to the corresponding author.
